# CLOCK inhibits the proliferation of porcine ovarian granulosa cells by targeting *ASB9*

**DOI:** 10.1186/s40104-023-00884-7

**Published:** 2023-06-07

**Authors:** Liang Huang, Huan Yuan, Shengjie Shi, Xiangrong Song, Lutong Zhang, Xiaoge Zhou, Lei Gao, Weijun Pang, Gongshe Yang, Guiyan Chu

**Affiliations:** 1 Key Laboratory of Animal Genetics, Breeding and Reproduction of Shaanxi Province, Yangling, 712100 China; 2 Laboratory of Animal Fat Deposition & Muscle Development, College of Animal Science and Technology, Northwest A&F University, Yangling, 712100 China

**Keywords:** ASB9, CLOCK, Granulosa cells, Pig, Proliferation

## Abstract

**Background:**

Clock circadian regulator (CLOCK) is a core factor of the mammalian biological clock system in regulating female fertility and ovarian physiology. However, CLOCK's specific function and molecular mechanism in porcine granulosa cells (GCs) remain unclear. In this study, we focused on CLOCK’s effects on GC proliferation.

**Results:**

CLOCK significantly inhibited cell proliferation in porcine GCs. CLOCK decreased the expression of cell cycle-related genes, including CCNB1, CCNE1, and CDK4 at the mRNA and protein levels. CDKN1A levels were upregulated by CLOCK. *ASB9* is a newly-identified target of CLOCK that inhibits GC proliferation; CLOCK binds to the E-box element in the *ASB9* promoter.

**Conclusions:**

These findings suggest that CLOCK inhibits the proliferation of porcine ovarian GCs by increasing *ASB9* level.

**Supplementary Information:**

The online version contains supplementary material available at 10.1186/s40104-023-00884-7.

## Introduction

Follicles are the functional unit of mammalian ovaries and are composed of membrane cells, granulosa cells (GCs), and oocytes [1]. GCs and oocytes are the most important cell types in the ovary [2]. GCs are the primary cell population in developing follicles that provide the necessary nutrients and microenvironment for oocytes during ovulation [3]. GCs are involved in critical physiological processes maintaining ovarian function, including proliferation, estradiol synthesis, and growth factor secretion [4, 5]. GC proliferation is essential for follicular development and oocyte maturation.

Circadian rhythm is an evolutionarily conserved time-keeping system with a periodicity of about 24 h; genetically encoded molecular clocks control it [6, 7]. Components of circadian clocks cooperate to regulate the physiology, biochemistry, and behavior of all organs for whole-body homeostasis in mammals [8]. Circadian clocks in all body cells depend on a transcriptional-translational feedback loop (TTFL) composed of clock genes [9]. Circadian locomotor output cycles kaput (*Clock*) is a core clock gene that encodes a transcription factor with a basic-helix-loop-helix PER-ARNT-SIM domain [10]. At the core of TTFL, CLOCK and aryl hydrocarbon receptor nuclear translocator-like (BMAL1) proteins in the cytoplasm form a heterodimeric transcription complex and bind to E-box elements (CANNTG) within the genes encoding the repressor proteins period (PER), cryptochrome (CRY), nuclear receptor subfamily 1 group D member 1(NR1D1), and retinoic acid orphan receptor α (RORα) [11]. Notably, circadian clocks are involved in reproductive processes and maintain mammalian fertility [12, 13]. Clock genes are expressed in the ovaries of several species, including humans [14], mice [15], rats [16], goats [17], and pigs [18]. Disruption of the circadian clock can affect fertility. *Per1*/*Per2* mutations significantly reduce the number of ovarian follicles and lead to premature depletion of ovarian follicle reserve and declining reproductive capacity in mice [19]. *Clock* knockdown of ovary impairs fertility in mice and reduces the release of oocytes and litter size [20]. Moreover, *Clock* small interfering RNA (siRNA) decreased the number of GCs cultured for 72 h in vitro in cattle [21]. These findings suggest a role for *CLOCK* in regulating GC function. However, the underlying mechanisms by which *CLOCK* regulates GC proliferation remain unclear, particularly in the pig.

Ankyrin repeat and suppressor of cytokine signaling (SOCS) box-containing 9 (ASB9) is a member of the eighteen members of the ASB family and is involved in many cellular processes, such as cell growth and differentiation [22, 23]. ASB9 interacts with mitochondrial creatine kinase to inhibit mitochondrial function and cell growth [24]. Previous studies demonstrated that ASB9 knockdown facilitated bovine ovarian GC proliferation by up-regulating *PCNA*, *CCND2*, and *CCNE2* levels [25]. However, the underlying mechanism that regulates the expression of *ASB9* in GCs is unknown. Since the E-box element exists in the promoter of *ASB9*, we hypothesized that CLOCK could directly regulate the expression of *ASB9*. In this study, transcriptome sequencing was performed after *CLOCK* overexpression in GCs. We found that *ASB9* is the target gene of CLOCK. Further studies revealed that CLOCK regulates GC proliferation by facilitating *ASB9* expression.

## Material and methods

### GCs isolation and culture

The Northwest Agriculture and Forestry University Animal Research Ethics Committee approved the use of animals and the experimental protocol (GB/T 35892–2018) [26]. Fresh Landrace (180-day-old and 110 kg) ovaries (*n* = 20) were collected from local slaughterhouses and stored in saline solution supplemented with penicillin and streptomycin (Cytiva, Shanghai, China) at 37 °C. The ovaries were transported to the laboratory within 2 h. Follicular fluid was extracted from healthy antral follicles (3–5 mm diameter) and centrifuged at 1,000 r/min for 10 min at room temperature. The pellets were washed with DEME/F12 (Cytiva, Shanghai, China) medium and centrifuged at 1,000 r/min for 5 min. Then, the pellets were resuspended in DEME/F12 medium containing 10% fetal bovine serum (ThermoFisher Scientific, Shanghai, China) and 1% penicillin–streptomycin (Cytiva, Shanghai, China). GCs were seeded at 4 × 10^5^/well in 6-well plates and 1 × 10^5^/well in 12-well plates and cultured in a 37 °C incubator with a humid atmosphere and 5% CO_2_. The information about the cell viability after the culture was shown in Additional file 1: Fig. S1. After 24 h of culture, the cells were gently washed with phosphate-buffered saline (PBS) (Cytiva, Shanghai, China), refreshed culture medium with dexamethasone (Sigma-Aldrich, Shanghai, China) at 100 nmol/L, and cultured for 2 h. The effect of dexamethasone is to synchronize the rhythm of GCs. Then, we replaced the medium. Finally, overexpression plasmid and siRNA were transfected into GCs.

### Immunofluorescence

GCs were cultured on 12-well plates and collected at 24 h. Briefly, the cells were washed with PBS, fixed with precooled 4% paraformaldehyde (Yike Biotechnology, Shaanxi, China) for 20 min, permeabilized with 0.5% TritonX-100 (Beyotime, Shanghai, China) for 10 min, and blocked with 5% bovine serum albumin (Servicebio, Wuhan, China) for 30 min. GCs were incubated with primary antibodies CLOCK (Abways, Shanghai, China) or FSHR (Abcam, Shanghai, China) at 37 °C for 2 h, then with anti-rabbit IgG (Boster, China, Wuhan) with fluorescence labels at room temperature for 2 h in the dark. Finally, GCs were stained with 4’,6-Diamidino-2’-phenylindole (DAPI) (Sigma-Aldrich, Shanghai, China) for 10 min, washed with PBS three times for 5 min, and photographed using a fluorescence microscope. The information of antibodies are displayed in Table 1. The negative controls of immunofluorescence was shown in Additional file 1: Fig. S2.

**Table 1:** The information of antibodies

Reagent type	Designation	Source	Catalog No.	Dilution rate/concentration
Antibody	GAPDH	Abways	AB0036	WB(1:5,000)
Antibody	CLOCK	Abways	CY6972	WB(1:1,000), IF(1:100)
Antibody	CCNB1	Abways	CY5378	WB(1:1,000)
Antibody	CCND1	Abways	CY5404	WB(1:1,000)
Antibody	CCNE1	Abways	CY1028	WB(1:1,000)
Antibody	CDK4	Abways	CY5836	WB(1:1,000)
Antibody	CDKN1A	Abways	CY5088	WB(1:1,000)
Antibody	ASB9	Santa Cruz	sc-166723	WB(1:1,000)
Antibody	HRP conjugated AffiniPure goat anti-mouse IgG (H + L)	Boster	BA1051	WB(1:5,000)
Antibody	HRP conjugated AffiniPure goat anti-rabbit IgG (H + L)	Boster	BA1054	WB(1:5,000)
Antibody	CY3 conjugated AffiniPure goat anti-rabbit IgG (H + L)	Boster	BA1032	IF(1:100)
Antibody	Anti-mouse IgG goat monoclonal antibody	Boster	M04575-3	ChIP (1μg)
Antibody	CLOCK	Santa Cruz	sc-271603	ChIP (1μg)

### RNA isolation and real-time quantitative PCR (RT-qPCR)

RNA isolation and RT-qPCR were performed as previously reported [27]. Total RNA samples were isolated using AG RNAex Pro RNA reagent (AG21101, Accurate Biotechnology (Hunan) Co., Ltd., Changsha, China), and the final concentrations were measured by NanoDrop 2000 (Thermo, Waltham, MA, USA). The cDNA was synthesized using the HiScript III RT SuperMix for qPCR (+ gDNA wiper) (Vazyme, Nanjing, China). The amount of RNA used for reverse transcription was 500 ng. RT-qPCR analysis of cDNA was performed using SYBR PCR mix (Vazyme, Nanjing, China) on a StepOne Real-Time PCR device (ABI, Carlsbad, CA, USA). The relative mRNA level was normalized to GAPDH and calculated using the 2^−ΔΔCt^ algorithm. The reaction conditions and the primer sequences used for RT-qPCR were displayed in Tables 2 and 3. The amplification efficiency of primers was shown in Additional file 1: Fig. S3. The negative controls in RT-qPCR was shown in Fig. S4.

**Table 2:** The reaction conditions of RT-qPCR

Stage	Cycle number	Temperature	Time
Predenaturation	1	95 °C	300 s
Denaturation	40	95 °C	10 s
Annealing	40	60 °C	30 s

**Table 3:** Primer sequences for real-time quantitative PCR

Gene name	Forward 5'→3'	Reverse 5'→3'	Size, bp	Accession No.
*GAPDH*	AGGTCGGAGTGAACGGATTTG	CCATGTAGTGGAGGTCAATGAAG	117	NM_001206359.1
*CLOCK*	GCCAGCAGCATGGTCCAGATTC	TCTGTCTGTCCTGAGGGAACGC	88	XM_021101324.1
*CCNB1*	AATCCCTTCTTGTGGTTA	CTTAGATGTGGCATACTTG	104	NM_001170768.1
*CCND1*	TACACCGACAACTCCATCCG	GAGGGCGGGTTGGAAATGAA	224	XM_021082686.1
*CCNE1*	AGAAGGAAAGGGATGCGAAGG	CCAAGGCTGATTGCCACACT	173	XM_005653265.2
*CDK1*	CAGCTCGCTACTCAACTCCA	GAGTGCCCAAAGCTCTGAAA	135	NM_001159304.2
*CDK4*	AAGTGGTGGGACAGTCAAGC	ACCACCACAGGTGTAAGTGC	81	NM_001123097.1
*ASB9*	ACCTGGGCACACCTTTATATTTGGC	GGTTCACACTCGCTCCTGATTCC	87	NM_001243703.1

### Western blot

Western blots were processed as previously reported [28]. Briefly, GCs were washed twice with pre-cooled PBS. Total proteins were collected using RIPA (Beyotime, Shanghai, China), and we added 120 μL RIPA to every well of 6-well plates supplemented with 1% protease inhibitors (CWBIO, Shanghai, China). We collected the cells into 1.5-mL centrifuge tubes and lysed them on ice for 30 min, then centrifuged (12,000 r/min) at 4 °C for 10 min. Protein concentrations were measured using a BCA protein assay kit (Thermo Fisher, Massachusetts, USA). A 1/4 volume of 5 × loading buffer (Ncmbio, Suzhou, China) was added to an aliquot of the supernatant and boiled for 10 min. Protein samples (20 μg/lane) were separated using 10% sodium dodecyl-polyacrylamide gel electrophoresis, and transferred protein to polyvinylidene fluoride membranes (CST, Boston, MA, USA) at 250 mA for 2.5 h. At room temperature, membranes were blocked with 5% skim milk for 2 h. Finally, the membranes were incubated with primary antibodies (1:1,000) to CLOCK, CCNB1, CCND1, CCNE1, CDK4, CDKN1A, GAPDH (Abways, Shanghai, China), or ASB9 (Santa Cruz, CA, USA) at 4 °C overnight and incubated with secondary antibody for 1 h at 4 °C. The secondary antibodies (1:5,000) were HRP goat anti-rabbit IgG and HRP goat anti-mouse IgG (BOSTER, Wuhan, China). The information of antibodies were displayed in Table 1. The negative controls of antibodies was shown in Fig. S5. The signals were detected using a chemiluminescence Western blotting substrate (Santa Cruz, CA, USA) and Image Lab analysis software Image Lab™ (Bio-Rad, Berkeley, CA, USA) and analyzed using Image J. All experiments were repeated at least three times, and mean values were calculated.

### Transient transfection of overexpression vector

Transfection of *CLOCK* or *ASB9* overexpression vector and control pcDNA3.1(+) (General Biol, Chuzhou, China) was performed using X-treme GENE HP DNA Transfection Reagent (Roche, Mannheim, Germany). Transfection was performed at 50% cell density, and samples were collected 24 h later.

### Transfection of siRNA

*CLOCK* siRNA, *ASB9* siRNA, and scrambled negative control were purchased from GenePharma (Shanghai, China) (Table 4). When the cell density reached 50%, the medium was removed, and the *CLOCK* or *ASB9* siRNA and nonsilencing RNA diluted in Opti-MEM reduced serum medium (Gbico, Shanghai, China) was transfected into cells using X-tremeGENE siRNA Transfection Reagent (Roche, Mannheim, Germany) according to the manufacturer's protocol. The *CLOCK* siRNA, *ASB9* siRNA, and nonsilencing RNA were used at final concentrations of 25 nmol/L. Samples were collected 24 h later.

**Table 4:** siRNA sequences targeting *CLOCK* and *ASB9* mRNA

Name	Target sequence 5'→3'	si-RNA sequence 5'→3'
si-CLOCK	GGGUCUGAUAAUCGUAUAA	F: GGGUCUGAUAAUCGUAUAATT
		R: UUAUACGAUUAUCAGACCCTT
si-ASB9	CCAUCCAUGAAGCUGCUAA	F: CCAUCCAUGAAGCUGCUAATT
		R: UUAGCAGCUUCAUGGAUGGTT

### Flow cytometry

GCs were cultured in 6-well plates at 4 × 10^5^ per well. GCs were treated with dexamethasone at 100 nmol/L for 2 h. The cells were treated with pcDNA3.1(+) plasmids, pcDNA3.1(+)-CLOCK plasmids, or pcDNA3.1(+)-ASB9 plasmids for 24 h and then digested with 0.25% trypsin and terminated with DMEM/F12 containing 10% fetal bovine serum. GCs were collected in 70% cold ethanol and fixed overnight at 4 °C. Finally, the cell cycle status of the GCs was analyzed using flow cytometry (Zimu, Shaanxi, China). PI/RNase Staining Buffer (BD Pharmingen™, New Jersey, USA) was used for flow cytometry. ModFit 3.0 was used to analyze the results.

### EdU assay

5-ethynyl-2′ deoxyuridine (EdU) assay was performed using a Cell-Light EdU Apollo567 In Vitro Kit (RiboBio, Guangzhou, China). Briefly, GCs were seeded in 96-well plates at 2 × 10^3^ per well. GCs were treated with dexamethasone at 100 nmol/L for 2 h. Then, GCs were treated with overexpression plasmids or siRNA for 24 h and incubated with 50 μmol EdU for 2 h. GCs were washed twice with PBS, fixed with 4% paraformaldehyde for 30 min, neutralized with 2 mg/mL glycine for 5 min, and then permeabilized with 0.5% TritonX-100 for 5 min. GCs were incubated in a mixture of reagents B, C, D, and E for 30 min. The cells were washed three times with 0.5% TritonX-100, followed by two washes with methanol. The nuclei were stained with Hoechst for 30 min. Finally, the cells were observed using a Nikon TE 2000 microscope (Nikon, Tokyo, Japan).

### Cell counting kit-8 (CCK-8)

The CCK-8 assay was performed using a kit according to the manufacturer’s instructions (Beyotime, Shanghai, China). GCs were seeded in 96-well plates with 2 × 10^3^ cells per well and treated with dexamethasone at 100 nmol/L for 2 h. Then, GCs were treated with overexpression plasmids or siRNA for 24 h. The cells were incubated with 10 μL CCK-8 reagent in an incubator at 37 °C for 3 h. Finally, absorbance was measured at 450 nm.

### Transcriptome sequencing

GCs were seeded in 6-well plates and treated with dexamethasone at a concentration of 100 nmol/L for 2 h. Then, GCs were treated with control and *CLOCK* overexpression plasmids for 24 h. Total RNA samples were isolated using AG RNAex Pro RNA reagent (AG21101, Accurate Biotechnology (Hunan) Co., Ltd., Changsha, China). According to a standard procedure, RNA sequencing was performed by Novogene Bioinformatics Technology Co., Ltd. (Beijing, China). RNA sequencing was completed based on Illumina. The total clean reads were mapped using NCBI Sus Scrofa RefSeq (Sscrofa 11.1). Differentially expressed genes (DEGs) were determined using DEseq2 software, and the genes with *P*-value < 0.05 and an absolute fold change > 1.0 were considered differentially expressed. The *P*-value (*P* < 0.05) was used to determine the significantly enriched GO terms and KEGG pathways. The information of RNA integrity number (RIN) was shown in Fig. S6. The number of reads in transcriptome sequencing was shown in Fig. S7. The raw data of transcriptome sequencing were deposited into the NCBI SRA database (BioProject ID: PRJNA944108).

### Luciferase reporter gene assay

We used the −1,664~−2,130 bp regions of the *ASB9* promoter to construct the wild-type (WT) vector and mutated the E-box sequences to construct the mutant (MUT) vector. We synthesized three fluorescent reporter plasmids: pGL3-ASB9 (WT), pGL3-ASB9 (MUT) and negative control pGL3-basic (General Biosystems, Anhui, China). Human embryonic kidney 293 (HEK293T) cells were seeded in 48-well plates. X-treme GENE HP DNA Transfection Reagent was used to transfect the luciferase reporter, pcDNA3.1(+), and pcDNA3.1(+)-CLOCK plasmids. The cells were harvested 24 h after transfection. Luciferase activities were measured using a Dual-Glo Luciferase Assay System (Promega; Madison, USA) following the manufacturer's instructions. Firefly luciferase was used as a normalization control.

### Chromatin immunoprecipitation (ChIP) assay

The ChIP assay was performed using a kit (Beyotime, Shanghai, China) according to the manufacturer’s instructions. Briefly, GCs were cross-linked with 1% formaldehyde at 37 °C. After washing with cold PBS containing phenylmethylsulfonyl fluoride, the cells were lysed in SDS Lysis Buffer contained phenylmethylsulfonyl fluoride. Chromatin was interrupted using an Ultrasonic Cell Disruption System (Sonics & Materials, Inc., USA) to generate 200–1,000 bp fragments. For the immunoprecipitation assay, the fragmented chromatin was incubatedwith anti-CLOCK (Santa Cruz, CA, USA), no antibody and mouse IgG at 4 °C overnight. No antibody and mouse IgG were used as the negative controls. After degrading proteins in the precipitated complexes with proteinase K, PCR was used to amplify the ChIP-purified DNA and input DNA. Finally, the amplified products were subjected to agarose gel electrophoresis and quantified with densitometry using ImageJ software. PCR amplifications were conducted using the* ASB9* promoter-specific primers as follows:

- *ASB9* promoter-forward: 5′-GAACAGTATGGAGGTTCCTC-3′, −1,989~ −1,966 bp relative to transcriptional start site.
- *ASB9* promoter-reverse: 5′-GGTTCTTGTGAAGAGTGCTG-3′, −2,115~ −2,096 bp relative to transcriptional start site.

### Statistical analysis

Experimental data were analyzed using GraphPad Prime 8. Results were expressed as mean ± SEM and included at least three independent samples. Two-tailed paired Student’s *t*-tests were used to compare two experimental groups. One-way and two-way ANOVA were used to compare three or more experimental groups. Dunnett and Sidak were used for post-hoc tests of one-way and two-way ANOVA, respectively. Statistically significant values of *P* < 0.05, *P* < 0.01, *P* < 0.001, and *P* < 0.0001 were indicated by *, **, ***, and ****, respectively.

## Results

### *CLOCK* is expressed in GCs and shows rhythmicity

**Fig. 1:**
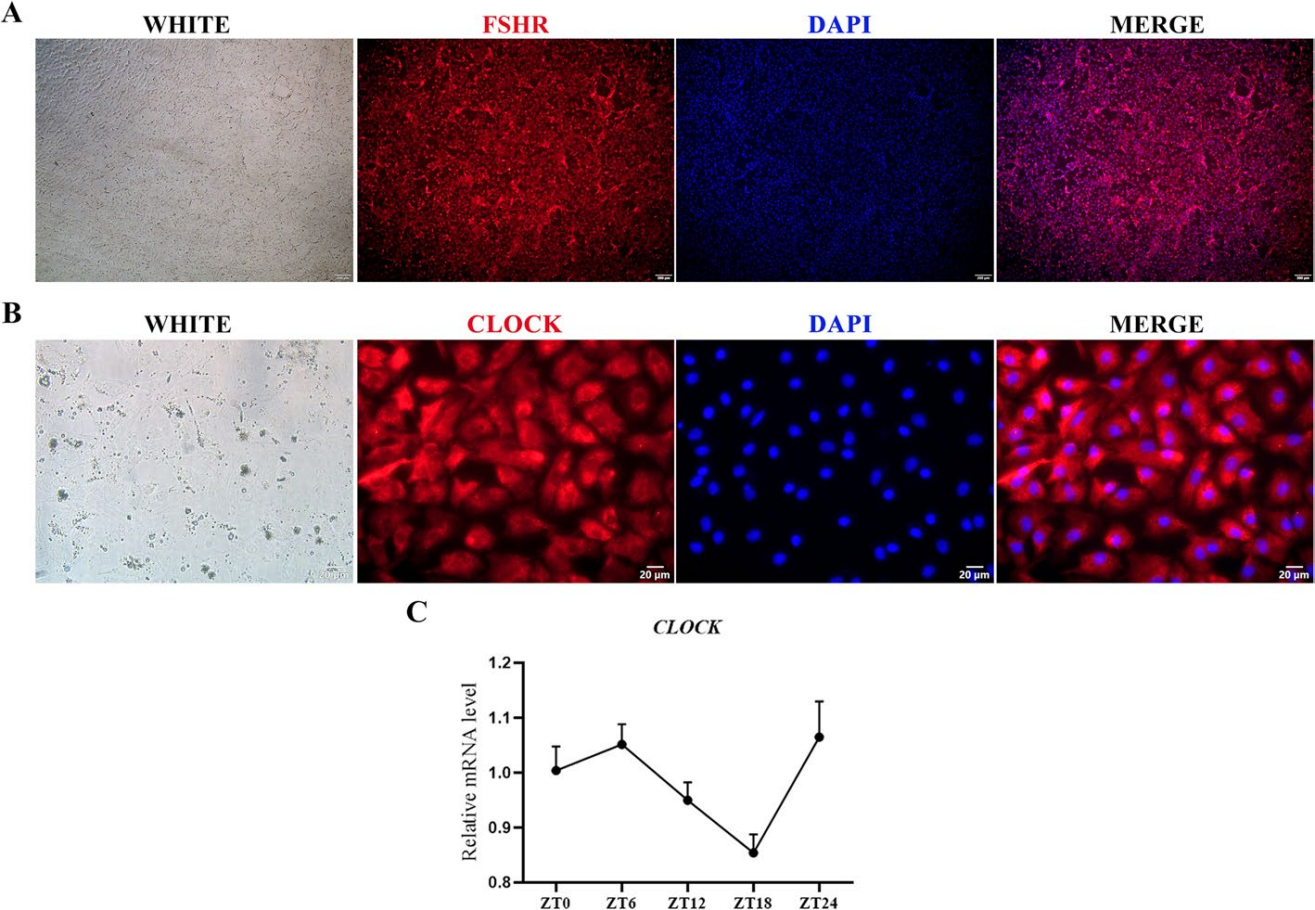
Localization and expression of circadian clock gene *CLOCK* in GCs. **A** Purity identification of cultured GCs in vitro. WHITE, white light; FSHR, red fluorescence; DAPI, blue fluorescence; bar = 200 µm. DAPI was used to visualize nuclei. **B** Immunofluorescence results reveal the expression of CLOCK in cultured GCs in vitro. WHITE, white light; CLOCK, red fluorescence; DAPI, blue fluorescence; bar = 20 µm. DAPI was used to visualize nuclei. **C** RNA expression of *CLOCK* in GCs. ZT: zone time

We identified the purity of primary GCs cultured in vitro, all of which carried red fluorescence of the specific receptor FSHR, indicating high purity (Fig. 1A). Cellular immunofluorescence staining was performed using a CLOCK-specific antibody. CLOCK was present in nuclei and cytoplasm of GCs (Fig. 1B). The expression patterns of *CLOCK* were explored after synchronizing with 100 nmol/L dexamethasone for 2 h. *CLOCK* was rhythmically expressed in porcine ovarian GCs (Fig. 1C).

### The role of CLOCK in GC proliferation

**Fig. 2:**
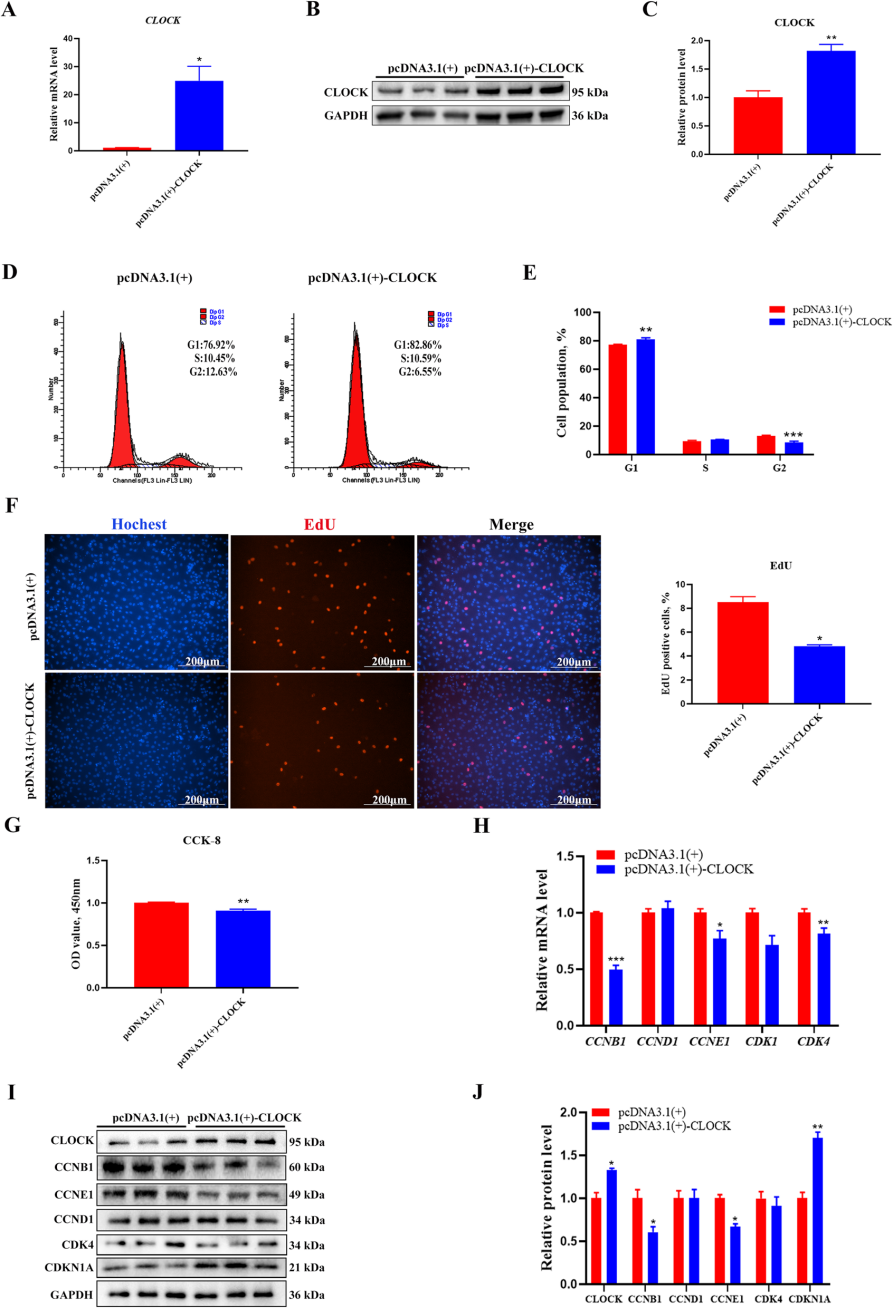
*CLOCK* overexpression inhibits GCs proliferation. **A** The overexpression efficiency of *CLOCK* was measured using RT-qPCR. Data are expressed as mean ± SEM (*n* = 4), ^*^P < 0.05. **B** Western blotting reveals the expression levels of CLOCK. **C** Quantitative statistics of CLOCK. Data are expressed as mean ± SEM (*n* = 3), ^**^P < 0.01. **D** Flow cytometry determines cell percentages in different cell-cycle phases. **E** Cell-cycle analysis statistical results. Data are expressed as mean ± SEM (*n* = 3), ^**^P < 0.01, ^***^P < 0.001. **F** EdU staining was used to quantify the number of proliferating cells. RED, EdU-positive cells; BLUE, Hoechst staining for total nuclei. Data are expressed as mean ± SEM (*n* = 3), ^*^P < 0.05. **G** CCK-8 assay detecting cell viability at 24 h after transfection. Data are expressed as mean ± SEM (*n* = 4), ^**^P < 0.01. **H** RT-qPCR analysis of proliferation-related genes, including *CCNB1*, *CCND1*, *CCNE1*, *CDK1*, and *CDK4*. Data are expressed as mean ± SEM (*n* = 3), ^*^P < 0.05, ^**^P < 0.01, ^***^P < 0.001. **I** Western blot analysis of proliferation-related gene protein level (CLOCK, CCNB1, CCND1, CCNE1, CDK4, and CDKN1A). GAPDH as a housekeeping protein. **J** Quantifying the Western blot analysis of CLOCK, CCNB1, CCND1, CCNE1, CDK4, and CDKN1A. Data are expressed as mean ± SEM (*n* = 3), ^*^P < 0.05, ^**^P < 0.01

**Fig. 3:**
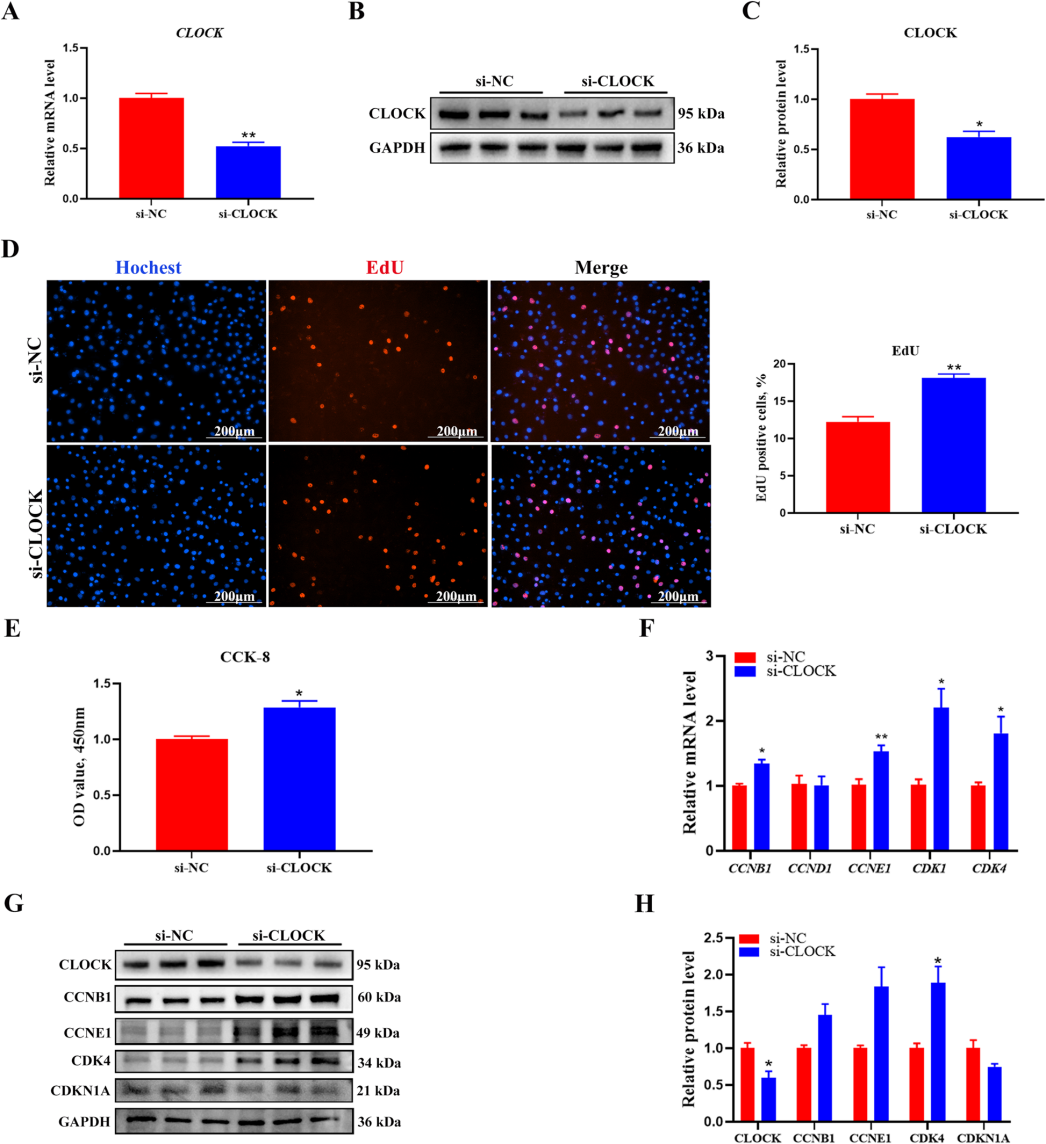
*CLOCK* interference promotes GCs proliferation. **A** The interference efficiency of *CLOCK* was measured using RT-qPCR. Data are expressed as mean ± SEM (*n* = 5), ^**^P < 0.01. **B** Western blotting reveals the expression levels of CLOCK. **C** Quantification of the western blot analysis. Data are expressed as mean ± SEM (*n* = 3), ^*^P < 0.05. **D** EdU staining was used to detect the number of proliferating cells. RED, EdU-positive cells; BLUE, Hoechst staining for total nuclei. Data are expressed as mean ± SEM (*n* = 5), ^**^P < 0.01. **E** CCK-8 assay detecting cell viability at 24 h after transfection. Data are expressed as mean ± SEM (*n* = 5), ^*^P < 0.05. **F** RT-qPCR analysis of proliferation-related genes, including *CCNB1*, *CCND1*, *CCNE1*, *CDK1*, and *CDK4*. Data are expressed as mean ± SEM (*n* = 5), ^*^P < 0.05, ^**^P < 0.01. **G** Western blot analysis of proliferation-related gene protein level (CLOCK, CCNB1, CCNE1, CDK4, and CDKN1A). GAPDH as a housekeeping protein. **H** Quantifying the Western blot analysis of CLOCK, CCNB1, CCNE1, CDK4, and CDKN1A. Data are expressed as mean ± SEM (*n* = 3), ^*^P < 0.05

The maturation of the follicle relies on GC proliferation, which is essential for maintaining reproductive function. To investigate the effect of the *CLOCK* gene on proliferation in GCs, we constructed an overexpression plasmid of *CLOCK* using the pcDNA3.1(+) vector. RT-qPCR showed that *CLOCK* was significantly increased at mRNA levels after transfection of *CLOCK* overexpression plasmid (Fold changes = 24.98) (Fig. 2A). As expected, the expression of CLOCK was also significantly increased (Fold changes = 1.82) (Fig. 2B, C). We determined the cell cycle distribution of GCs using flow cytometry. CLOCK significantly increased the number of cells in the G1-phase and decreased the number of cells in the G2-phase (Fig. 2D, E). The EdU-staining assay revealed that there were fewer EdU-labeled cells in CLOCK overexpression-treated cells than in control-treated cells (Fig. 2F). The CCK-8 assay revealed that CLOCK inhibited the viability of GCs (Fig. 2G). RT-qPCR revealed that *CLOCK* overexpression significantly inhibited the expression of proliferation-related genes, including *CCNB1*, *CCNE1*, and *CDK4* (Fig. 2H). The protein levels of CCNB1 and CCNE1 were significantly downregulated (Fig. 2I, J), and CDKN1A levels increased (Fig. 2I, J). We suppressed the expression of CLOCK at mRNA (Fold changes = 0.52) and protein (Fold changes = 0.62) levels by transfecting with siRNA (Fig. 3A–C). Interference with *CLOCK* increased the number of the EdU-positive GCs (Fig. 3D). The CCK-8 assay showed that CLOCK significantly promoted the viability of GCs (Fig. 3E). CLOCK siRNA markedly increased mRNA levels of *CCNB1*,* CCNE1*, *CDK1*, and* CDK4* (Fig. 3F). Similarly, CDK4 protein levels were significantly increased (Fig. 3G, H). In addition, CCNB1 and CCNE1 were also increased but no significant (Fig. 3G, H). These findings suggest that CLOCK inhibits proliferation in cultured GCs.

### CLOCK regulates *ASB9* expression in GCs

**Fig. 4:**
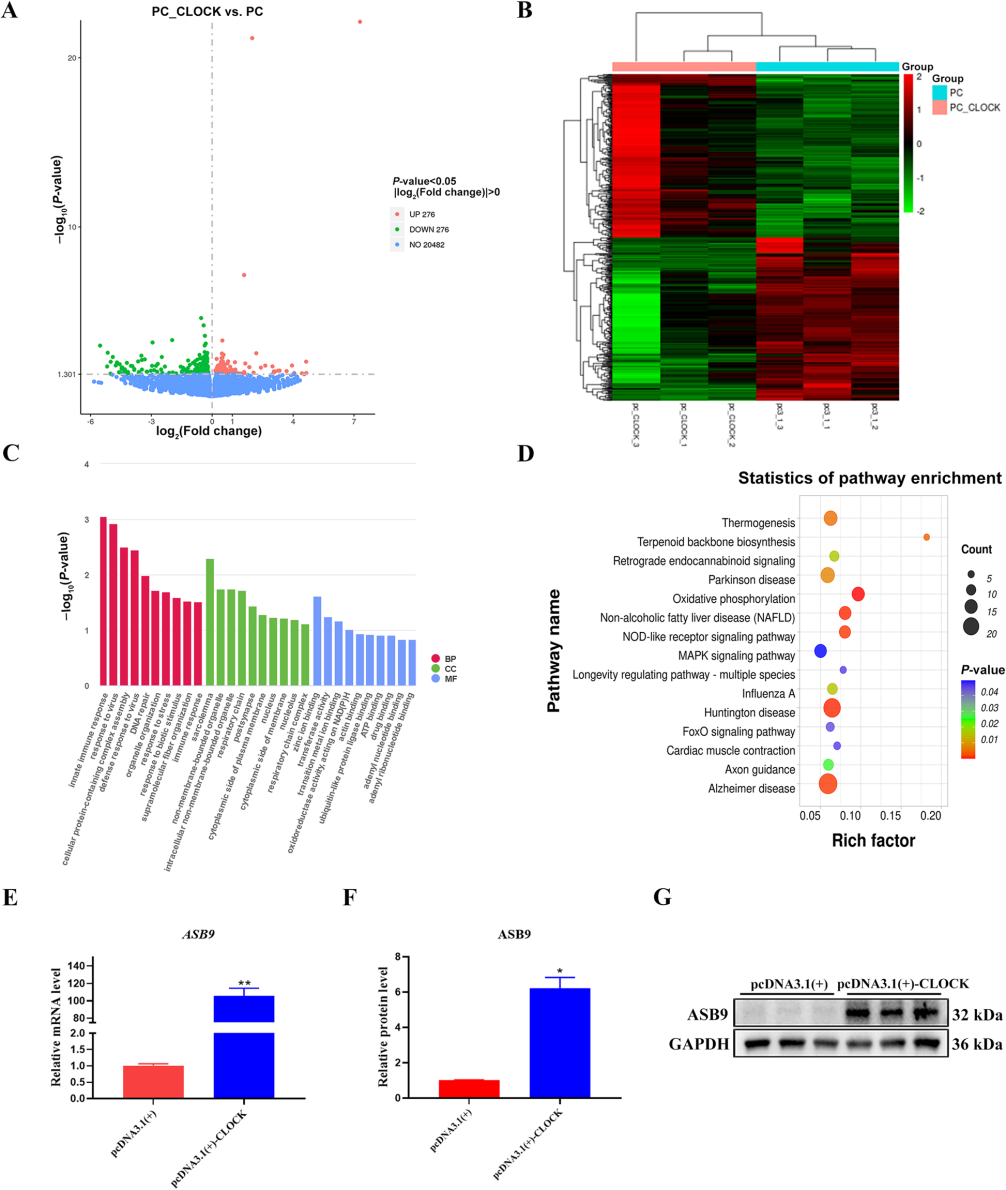
Transcriptomic profiling of GCs with *CLOCK* overexpression treatment. **A** A volcano plot of the expressed genes. **B** Heatmap of the differentially expressed genes in GCs overexpressing *CLOCK* according to RNA-seq. **C** Gene ontology analysis. BP: biological process; CC: cellular component; MF: molecular function. **D** Kyoto Encyclopedia of Genes and Genomes pathway analysis. **E** RT-qPCR detected the expression levels of *ASB9*. Data are expressed as mean ± SEM (*n* = 3), ^**^P < 0.01. **F** Quantitative statistics of ASB9. Data are expressed as mean ± SEM (*n* = 3), ^*^P < 0.05. **G** Western blotting revealed the expression levels of ASB9

To determine CLOCK's associated pathways and potential targets in GCs, RNA sequencing (RNA-seq) was performed in *CLOCK*-overexpressing GCs, with GCs expressing pcDNA3.1(+) as a control. Principal components analysis (PCA) was shown in Fig. S8. We analyzed 21,034 genes, and 552 genes were differentially expressed, among which 276 genes were upregulated and 276 were downregulated, as shown in the volcano plot (Fig. 4A). A heatmap was used to visualize the differential gene expression (Fig. 4B). We performed Gene Ontology analysis on the differential genes in GCs. We found that CLOCK participates in various biological processes, such as innate immune response, cellular protein-containing complex assembly, and response to virus, and plays different roles by regulating target genes (Fig. 4C). Kyoto Encyclopedia of Genes and Genomes pathway analysis revealed that it was significantly enriched in FoxO signaling pathway and MAPK signaling pathway related to cell proliferation (Fig. 4D). Notably, we observed that *ASB9* was the most significant of the differentially expressed genes (Additional file 2: Table S1). RT-qPCR and western blot revealed that *CLOCK* overexpression dramatically increased ASB9 expression at the mRNA and protein levels (Fig. 4E–G). We hypothesized that ASB9 has crucial function in porcine ovarian GCs.

### ASB9 affects the function of GCs

**Fig. 5:**
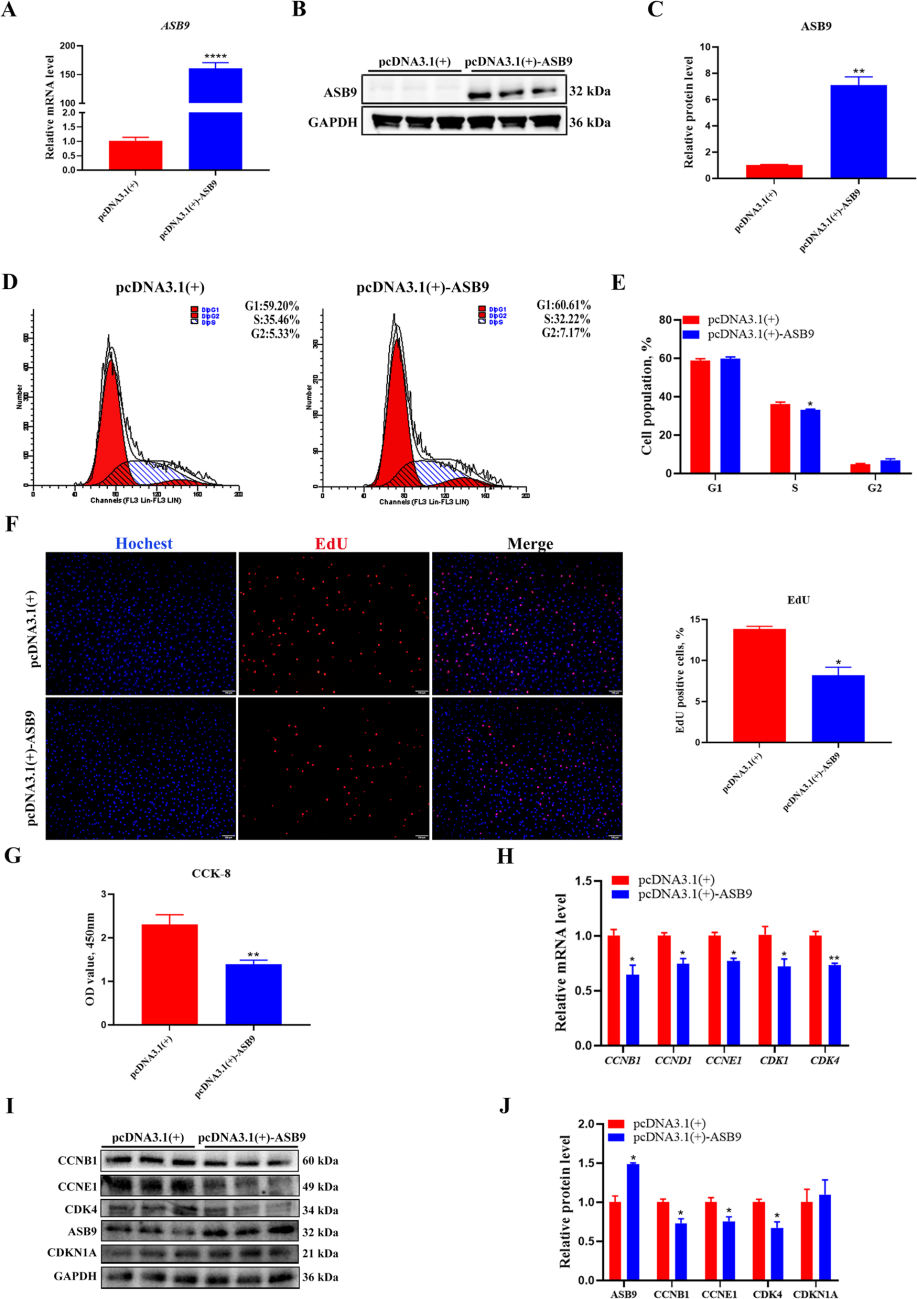
*ASB9* overexpression inhibits GCs proliferation. **A** The overexpression efficiency of *ASB9* was detected using RT-qPCR. Data are expressed as mean ± SEM (*n* = 6), ^****^P < 0.0001. **B** Western blotting reveals the expression levels of ASB9. **C** Quantitative statistics of ASB9. Data are expressed as mean ± SEM (*n* = 3), ^**^P < 0.01. **D** Flow cytometry determines cell percentages in different cell cycle phases. **E** Cell cycle analysis statistical results. Data are expressed as mean ± SEM (*n* = 4), ^*^P < 0.05. **F** EdU staining was used to detect the number of proliferating cells. RED, EdU-positive cells; BLUE, Hoechst staining for total nuclei. Data are expressed as mean ± SEM (*n* = 3), ^*^P < 0.05. **G** CCK-8 assay detecting cell viability at 24 h after transfection. Data are expressed as mean ± SEM (*n* = 16), ^**^P < 0.01. **H** RT-qPCR analysis of proliferation-related genes, including *CCNB1*, *CCND1*, *CCNE1*, *CDK1*, and *CDK4*. Data are expressed as mean ± SEM (*n* = 6), ^*^P < 0.05, ^**^P < 0.01. **I** Western blot analysis of proliferation-related gene protein level (ASB9, CCNB1, CCNE1, CDK4, and CDKN1A). **J** Quantifying the western blot analysis of ASB9, CCNB1, CCNE1, CDK4, and CDKN1A. Data are expressed as mean ± SEM (*n* = 3), ^*^P < 0.05

**Fig. 6:**
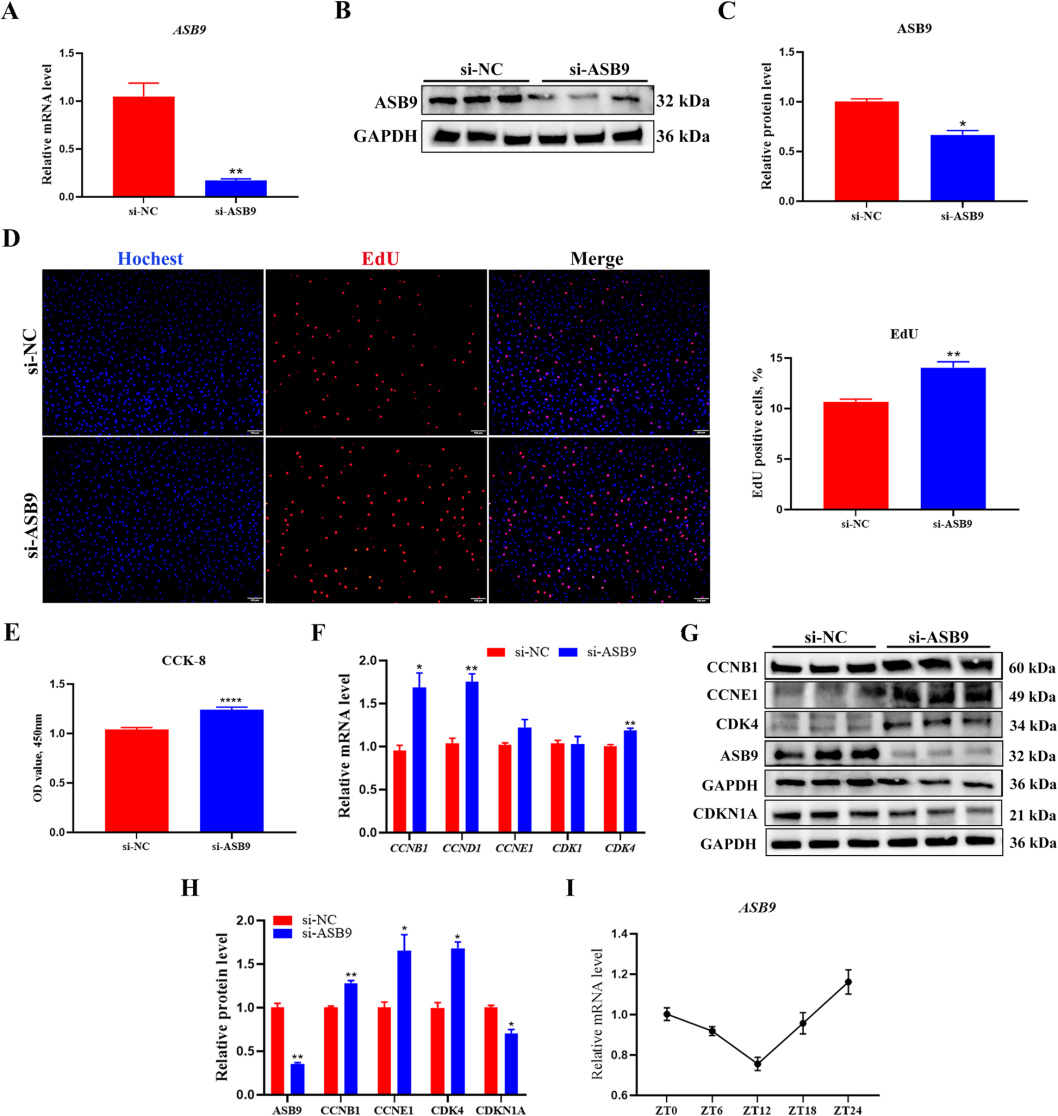
*ASB9* interference promotes GCs proliferation. **A** RT-qPCR detected the interference efficiency of *ASB9*. Data are expressed as mean ± SEM (*n* = 6), ^**^P < 0.01. **B** Western blotting reveals the expression levels of ASB9. **C** Quantification of the western blot analysis. Data are expressed as mean ± SEM (*n* = 3), ^*^P < 0.05. **D** EdU staining was used to detect the number of proliferating cells. RED, EdU-positive cells; BLUE, Hoechst staining for total nuclei. Data are expressed as mean ± SEM (*n* = 4), ^**^P < *0.01*. **E** CCK-8 assay detecting cell viability at 24 h after transfection. Data are expressed as mean ± SEM (*n* = 16), ^****^P < 0.0001. **F** RT-qPCR analysis of proliferation-related genes, including *CCNB1*, *CCND1*, *CCNE1*, *CDK1*, and *CDK4*. Data are expressed as mean ± SEM (*n* = 5), ^*^P < 0.05, ^**^P < 0.01. **G** Western blot analysis of proliferation-related gene protein level (ASB9, CCNB1, CCNE1, CDK4, and CDKN1A). **H** Quantifying the western blot analysis of CLOCK, CCNB1, CCNE1, CDK4, and CDKN1A. Data are expressed as mean ± SEM (*n* = 3), ^*^P < 0.05, ^**^P < 0.01. **I** RNA expression of *ASB9* in GCs. ZT: zone time

To test our hypothesis, we investigated the role of ASB9 in GC proliferation. *ASB9* was overexpressed or silenced using pcDNA3.1(+)-ASB9 plasmids or ASB9-directed siRNA. RT-qPCR and Western blot analyses showed that the ASB9 was remarkably increased at mRNA (Fold changes = 160.88) and protein (Fold changes = 7.10) levels (Fig. 5A–C). Flow cytometry showed that ASB9 significantly decreased the number of cells in the S-phase in GCs (Fig. 5D, E). Cell proliferative capacity was evaluated using an EdU assay. As expected, the decreased proportion of EdU-positive cells confirmed the inhibited cell proliferation by *ASB9* overexpression (Fig. 5F). The CCK-8 assay indicated that cell viability was significantly impaired by *ASB9* overexpression (Fig. 5G). RT-qPCR revealed that *ASB9* overexpression significantly inhibited the expression of proliferation-related genes, including *CCNB1*, *CCND1*, *CCNE1*, *CDK1*, and *CDK4* (Fig. 5H). Protein levels of CCNB1, CCNE1, and CDK4 were significantly downregulated (Fig. 5I, J). In addition, we suppressed the expression of *ASB9* at mRNA (Fold changes = 0.17) and protein (Fold changes = 0.67) levels by transfecting with siRNA (Fig. 6A–C). *ASB9* knockdown significantly increased the number of EdU-positive cells and enhanced GCs viability (Fig. 6D, E). *ASB9* knockdown increased *CCNB1*, *CCND1*, and *CDK4* at mRNA levels (Fig. 6F). Protein levels of CCNB1, CCNE1, and CDK4 were significantly upregulated (Fig. 6G, H). CDKN1A levels decreased (Fig. 6G, H). These findings suggest that ASB9 inhibits GC proliferation. In addition, we checked the expression (rhythm) of *ASB9*. *ASB9* was rhythmically expressed at mRNA level in GC (Fig. 6I).

### *ASB9* is a direct target of CLOCK in GCs

**Fig. 7:**
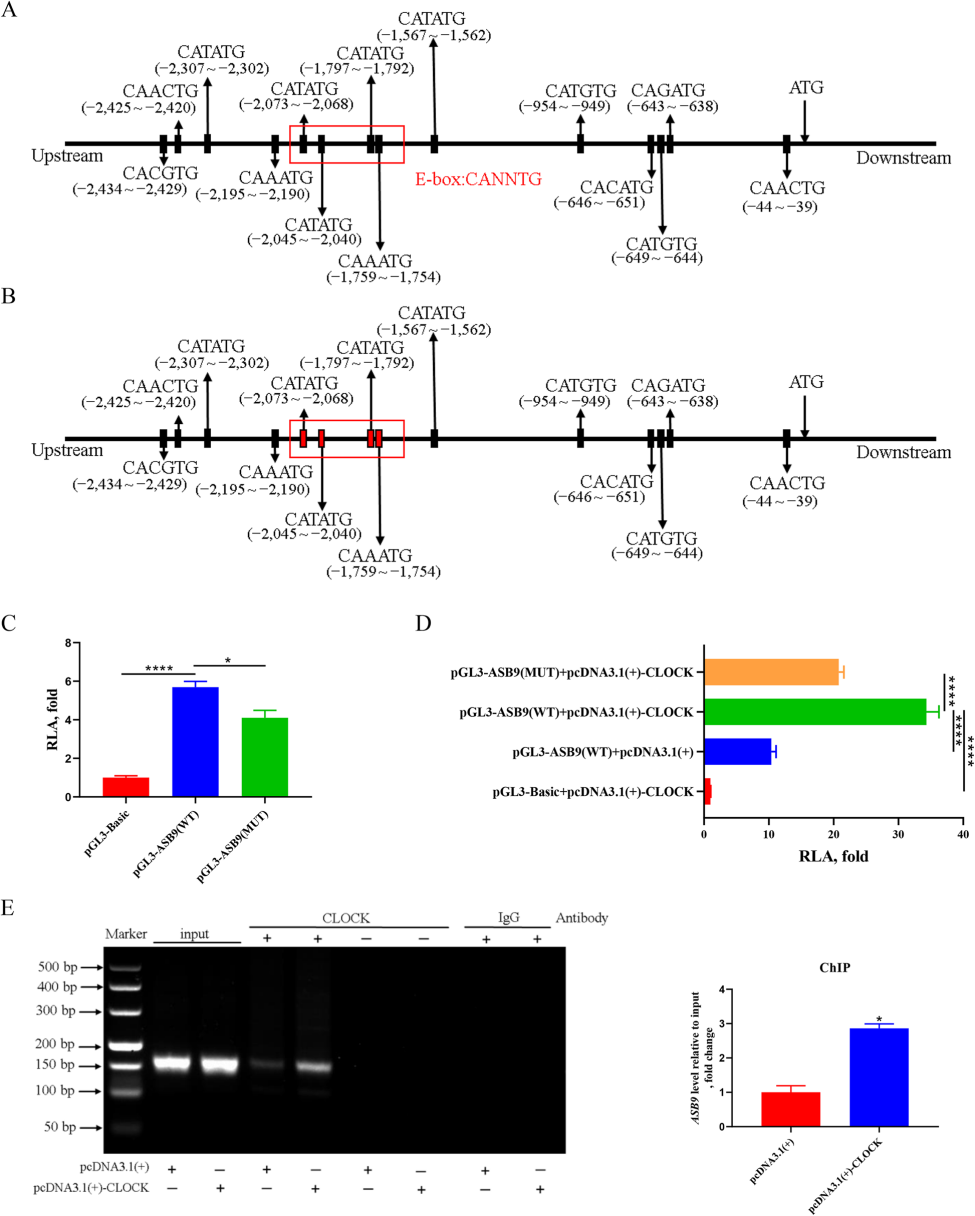
CLOCK promotes the expression of *ASB9* in GCs. **A** Sequence analysis of E-box on *ASB9* promoter. **B** Mutated E-box sequences of the luciferase reporter. **C** and **D** Luciferase reporter assays of ASB9‐Luc reporter constructs. The wild-type and the mutant pGL3-ASB9 reporters were co-transfected into HEK293T cells with pcDNA3.1(+)/pcDNA3.1(+)-CLOCK plasim, and the fluorescence activity was detected 24 h later. Data are expressed as mean ± SEM (*n* = 5), ^*^P < 0.05, ^****^P < 0.0001. **E** ChIP assay showing recruitment of CLOCK protein to *ASB9* E‐box in GCs. The PCR products were analysed on a 2% agarose gel and quantified with densitometry using ImageJ software. IgG and no antibody were used as the negative ChIP control. Data are expressed as mean ± SEM (*n* = 3), ^*^P < 0.05

To determine whether CLOCK directly regulates the expression of *ASB9*, we analyzed the pre-2,500 bp sequence of the start codon ATG of *ASB9*. Sequence analysis revealed fourteen potential E-box (CANNTG) elements (Fig. 7A). We used the BDZF online tool to predict the active region of the *ASB9* promoter (−1,732 – −1,782 bp) and then constructed luciferase reporter from the −1,664 – −2,130 bp regions (Fig. 7B). In luciferase reporter assays, CLOCK significantly increased *ASB9* promoter activity (Fig. 7C, D). ChIP assays showed that higher amounts of CLOCK were associated with the *ASB9* promoter in overexpressing *CLOCK* in GCs (Fig. 7E). These findings suggest that CLOCK-mediated transcriptional activation of *ASB9* occurs via direct binding to E‐box elements in promoter regions. To confirm a direct association between CLOCK and ASB9, we performed co-transfection experiments to see the effect of CLOCK overexpression on GC-depleting ASB9 function. We found that the overexpression of CLOCK together with inhibition of ASB9 could restore CDK4 protein level and remove the inhibition of CDKN1A (Fig. S9).

## Discussion

*CLOCK* is a core circadian clock gene that has been heavily studied in several organs and cells [29–31]. Several lines of evidence suggest that circadian clocks play a crucial role in regulating ovarian function [32–34]. Circadian clock genes perform various roles in regulating different physiological processes in the ovaries [16, 35, 36]*.* Although the *CLOCK* gene has been studied to some extent in humans and mice [14, 20], there has been little research on porcine ovaries. Previous studies demonstrated that CLOCK was expressed in the cumulus cell and mural GC of dominant antral follicles but not in preantral follicles in the human ovary [34]. In the present study, CLOCK inhibited GC proliferation by increasing *ASB9* level. Our findings provide insights into the effects of CLOCK on GC proliferation.

Follicular development and oocyte maturation rely on GC proliferation [37, 38]. Proliferating GCs support somatic cells in follicles, controlling the progression of folliculogenesis and providing the microenvironment required for acquiring a meiotically competent oocyte [39, 40]. GC proliferation is regulated by a complex network consisting of several factors [41]. The findings in the present study suggest that CLOCK inhibits GC proliferation by downregulating CCNB1 and CCNE1 at the mRNA and protein levels. Cyclin E and Cyclin B are crucial regulators of the cell cycle, controlling the G1/S transition and the G2/M transition, respectively [42], which could explain the cell phenotype. Our research differ from a previous study in cattle [21]. In that study, there is effect of CLOCK siRNA on cell number at 72 h but not 24 h [21]. There are several reasons can explain this discrepancy. On the one hand, it might be species specificity because the amino acids of CLOCK from pigs and cattles differ. On the other hand, the condition of culture medium and experimental methods in our experiment are different from the previous study. This could lead to different experimental results. Moreover, CLOCK promotes or inhibits cell proliferation and varies across cell types and species [43–47]. For example, CLOCK knockdown increases CCND1 level and promotes the growth of mammary epithelial cells in mice [43]. CLOCK promotes HeLa cell proliferation via RHOA protein [44]. Clock overexpression suppressed cell growth in human colon cancer cells [45] and increased G0/G1 phase cells in ovarian cancer SKOV3/DDP cells [46]. Clock silencing decreased cell proliferation rate by reducing *C-myc*, *CDK4*, and *CyclinD1* levels in mouse embryonic stem cells [47].

ASB9 is a member of the most prominent family of SOCS box-containing superfamily proteins and is an E3 ubiquitin ligase [48]. A conserved SOCS box motif and a variable number of ankyrin repeats characterize ASB9 [49]. E3 ligases catalyze the highly-specific covalent attachment of activated ubiquitin to substrate proteins through an isopeptide bond on an exposed lysine residue [50, 51]. ASB9 plays a vital role in protein ubiquitination. To characterize the molecular mechanisms by which CLOCK regulates GC proliferation, we performed RNA-seq in *CLOCK* overexpression-treated GCs. There were 552 differentially expressed genes, among which 276 genes were upregulated and 276 were downregulated. Gene Ontology analysis showed that CLOCK participates in various biological processes, such as innate immune response, cellular protein-containing complex assembly, and DNA repair. We selected *ASB9*, which is the most differentially expressed gene. Our results suggest that ASB9 inhibits cell proliferation in porcine GCs, which is concent with the study in which ASB9 inhibition increases GC number by regulating cell cycle related genes, including *PCNA*, *CCND2*, and *CCNE2* [25]. These findings suggest that ASB9 is involved in regulating cell proliferation.

The heterodimeric transcriptional activators CLOCK and BMAL1 promote the transcription of clock-controlled genes by binding E-box elements in the promoter region [52]. Circadian clock genes are associated with the cell cycle and modulate cellular proliferation. Previous studies showed that clock proteins regulate cell cycle progression by binding to E-box or RRE elements on target gene promoters, such as WEE1, c-MYC, and p21 [11, 53–57]. In the present study, the double luciferase reporter gene and ChIP assays confirmed *ASB9* as a direct CLOCK target. We found that CLOCK positively regulates *ASB9* at the transcriptional level by binding the E-box domain, suggesting that CLOCK affects GC proliferation by regulating the expression of *ASB9*.

## Conclusions

**Fig. 8:**
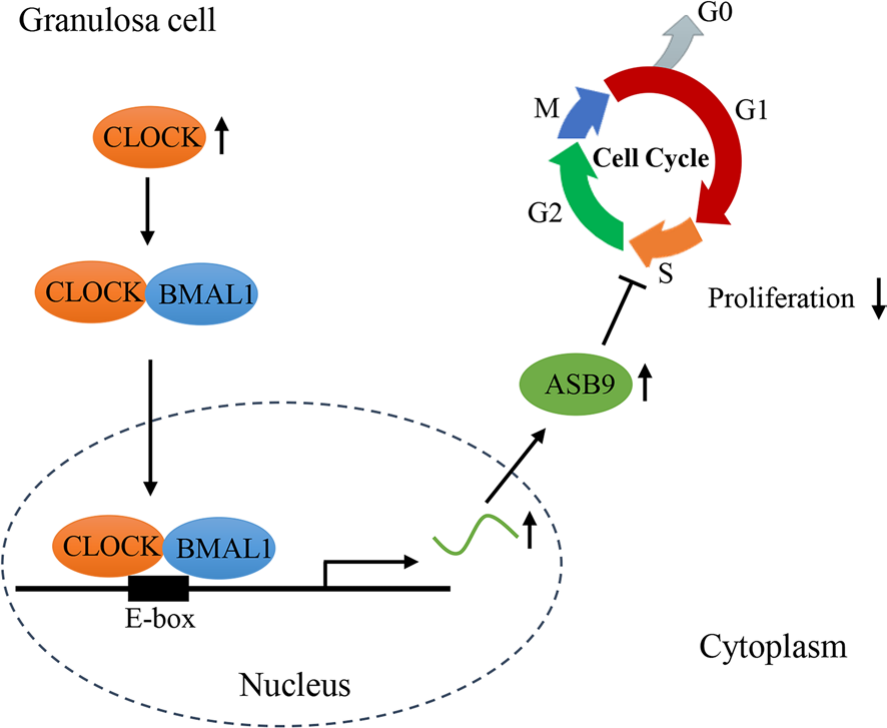
Schematic diagram of CLOCK regulation on porcine GC proliferation. CLOCK inhibits cell proliferation by promoting *ASB9* expression in porcine ovarian GCs. Specifically, CLOCK and BMAL1 complex binds to the E-box element of *ASB9* promoter to increase the level of *ASB9*. Then, ASB9 inhibits GC proliferation by regulating cell cycle

We identified a critical role of CLOCK in regulating GC proliferation (Fig. 8). CLOCK inhibits cell proliferation by promoting *ASB9* expression in porcine ovarian GCs. These findings provide insights into the biological function of CLOCK in modulating GC proliferation. This study indicate that circadian rhythms is very important to maintain normal reproductive function through circadian control genes.

## Supplementary Information

**Additional file 1:** **Fig. S1.** The information about the cell viability after the culture. **Fig. S2.** The negative controls of immunofluorescence. **Fig. S3.** The amplification efficiency of primers in RT-qPCR. **Fig. S4.** The negative controls in RT-qPCR. **Fig. S5.** The negative controls of antibodies in western blot. **Fig. S6.** The information of RNA integrity numberin transcriptome sequencing. **Fig. S7.** The number of reads in transcriptome sequencing. **Fig. S8.** Principal components analysisin transcriptome sequencing. **Fig. S9.** A direct association between CLOCK and ASB9 using co-transfection experiments.

**Additional file 2:** **Table S1.** Differentially expressed genes.

## Data Availability

The data sets used and analyzed during the current study are available from the corresponding author on reasonable request.

## Abbreviations

ASB9: Ankyrin repeat and suppressor of cytokine signaling (SOCS) box-containing 9
CCNB1: Cyclin B1
CCND1: Cyclin D1
CCNE1: Cyclin E1
CDK1: Cyclin dependent kinase 1
CDK4: Cyclin dependent kinase 4
CDKN1A: Cyclin dependent kinase inhibitor 1A
ChIP: Chromatin Immunoprecipitation
CLOCK: Clock circadian regulator
EdU: 5-ethynyl-2′ deoxyuridine
GCs: Granulosa cells
TTFL: Transcriptional-translational feedback loop
