# Supplementary material for: CLOCK inhibits the proliferation of porcine ovarian granulosa cells by targeting ASB9
Source: J Anim Sci Biotechnol. 2023 Jun 7;14:82. doi: 10.1186/s40104-023-00884-7 (PMC10245596; doi:10.1186/s40104-023-00884-7)
Supplement: Supplementary file 1 — Additional file 1: Fig. S1. The information about the cell viability after the culture. Fig. S2. The negative controls of immunofluorescence. Fig. S3. The amplification efficiency of primers in RT-qPCR. Fig. S4. The negative controls in RT-qPCR. Fig. S5. The negative controls of antibodies in western blot. Fig. S6. The information of RNA integrity numberin transcriptome sequencing. Fig. S7. The number of reads in transcriptome sequencing. Fig. S8. Principal components analysisin transcriptome sequencing. Fig. S9. A direct association between CLOCK and ASB9 using co-transfection experiments. [file 40104_2023_884_MOESM1_ESM.docx]

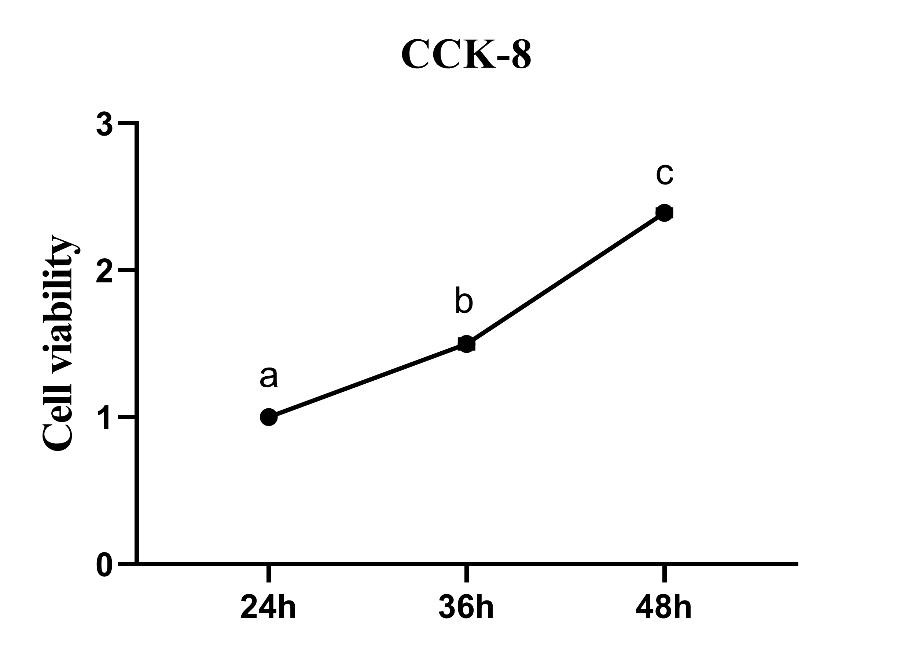


**Fig. S1** The information about the cell viability after the culture


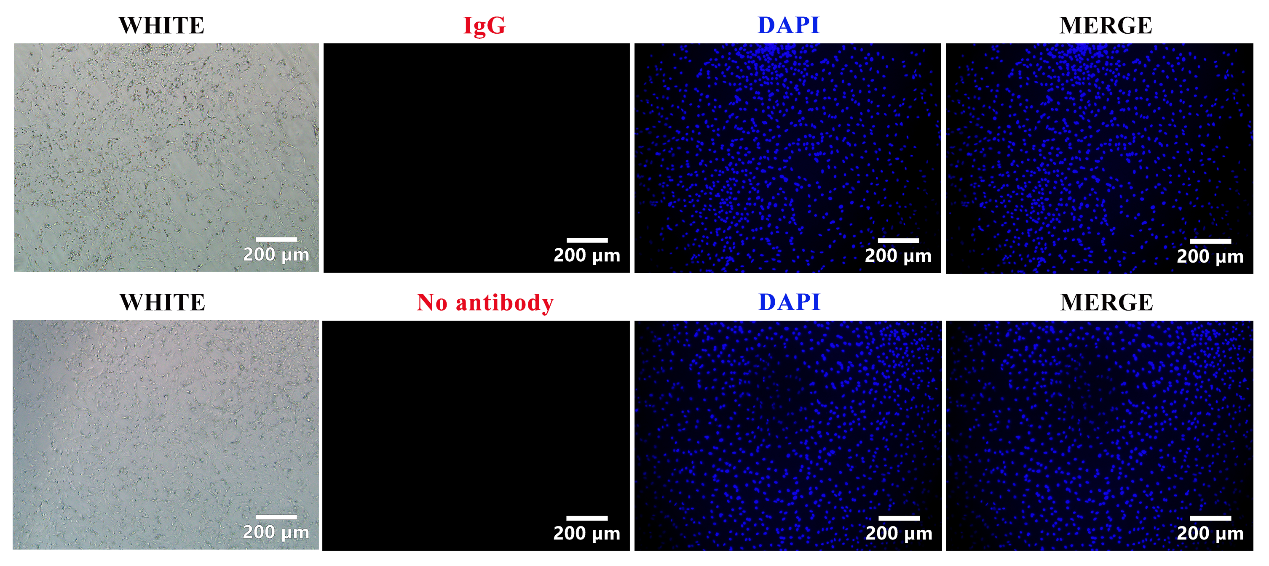


**Fig. S2** The negative controls of immunofluorescence


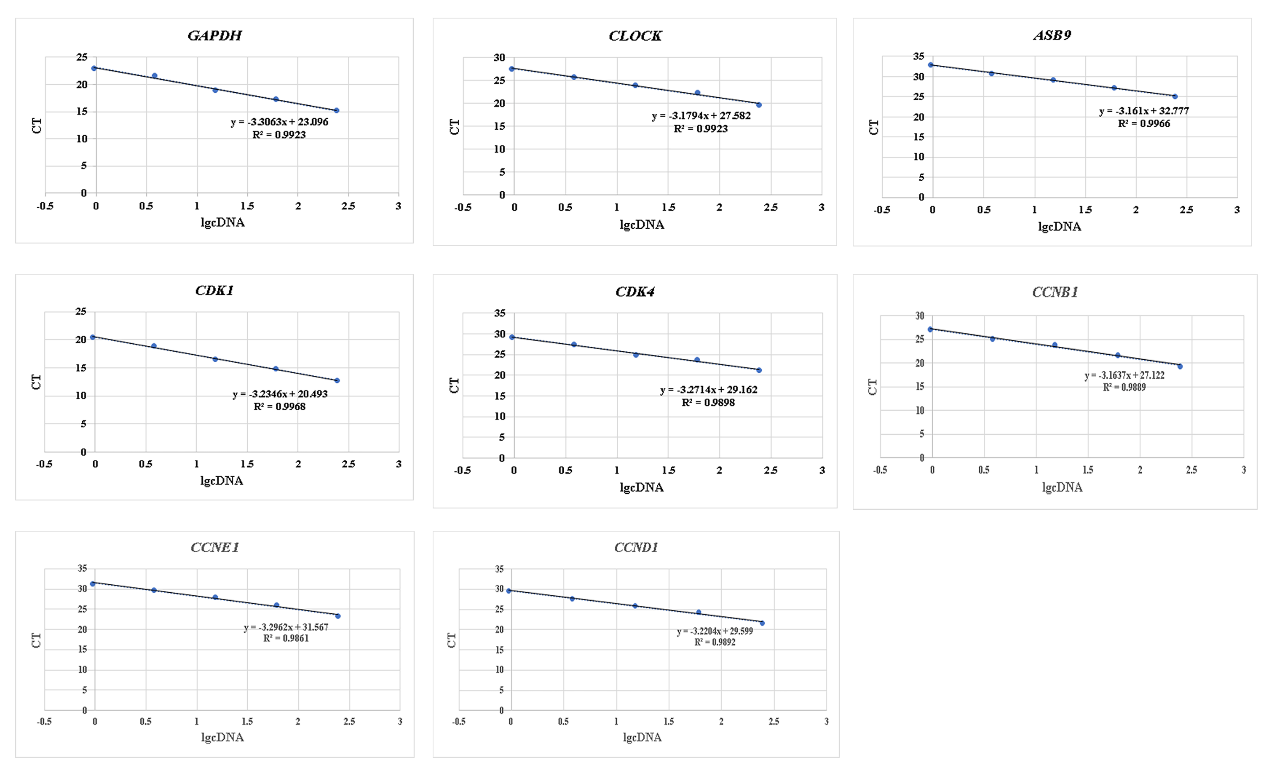


**Fig. S3** The amplification efficiency of primers in RT-qPCR


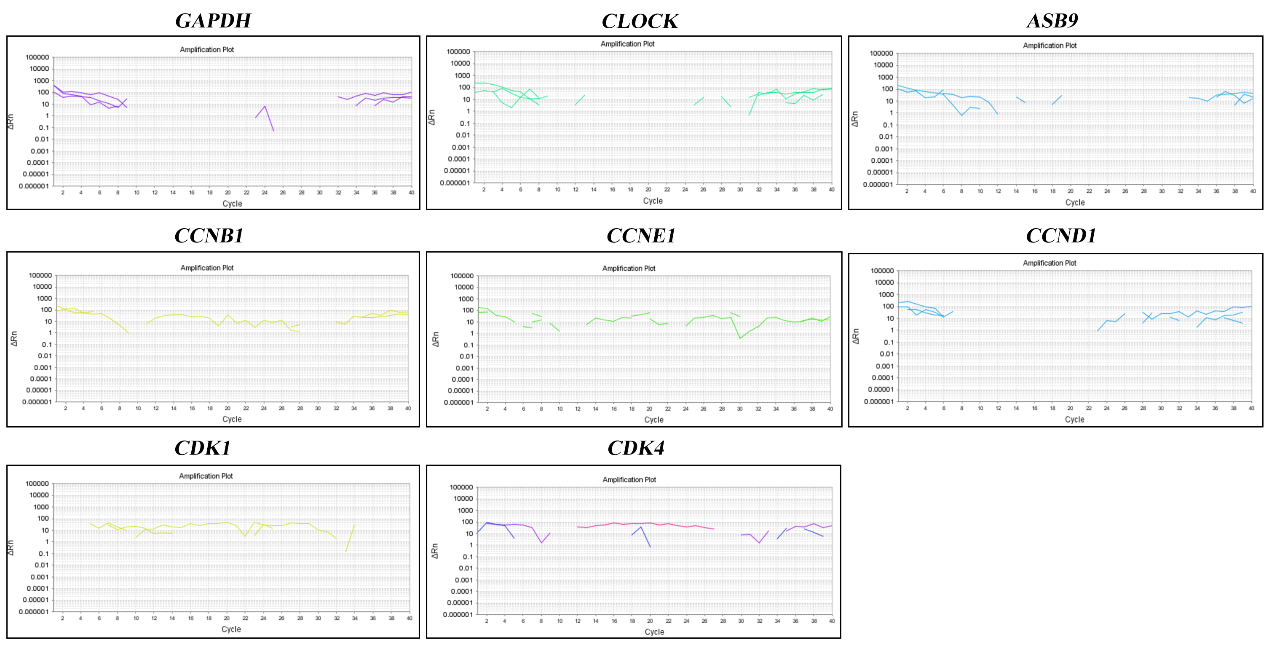


**Fig. S4** The negative controls in RT-qPCR


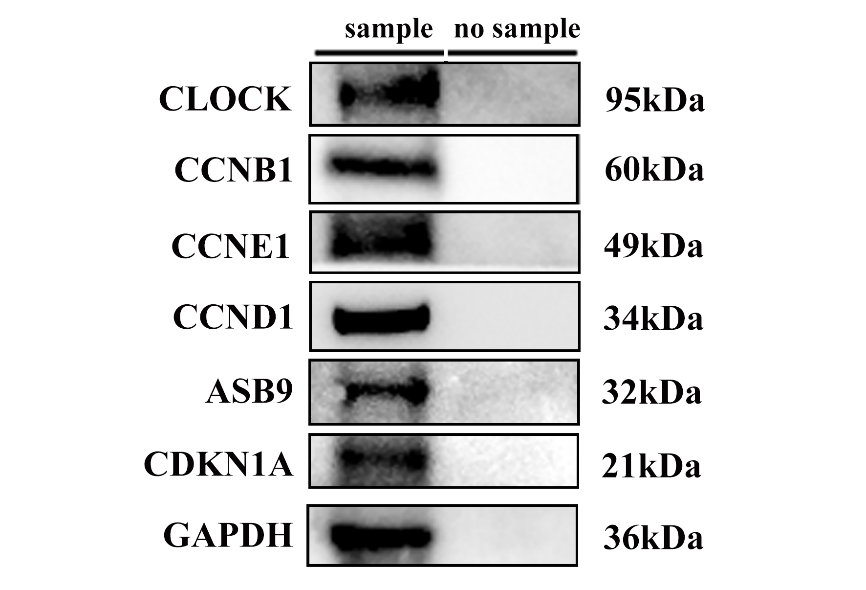


**Fig. S5** The negative controls of antibodies in Western blot


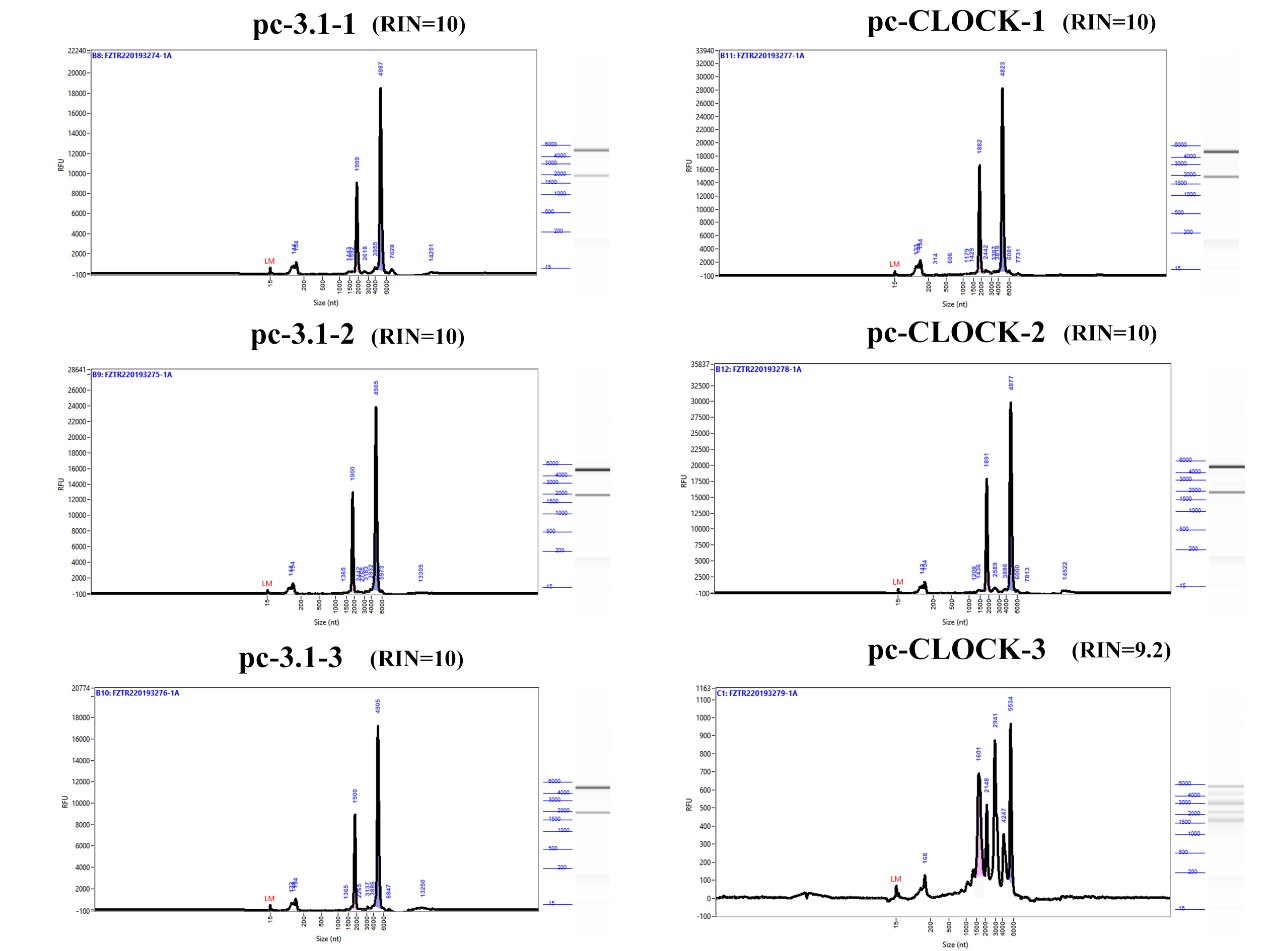


**Fig. S6** The information of RNA integrity number (RIN) in transcriptome sequencing


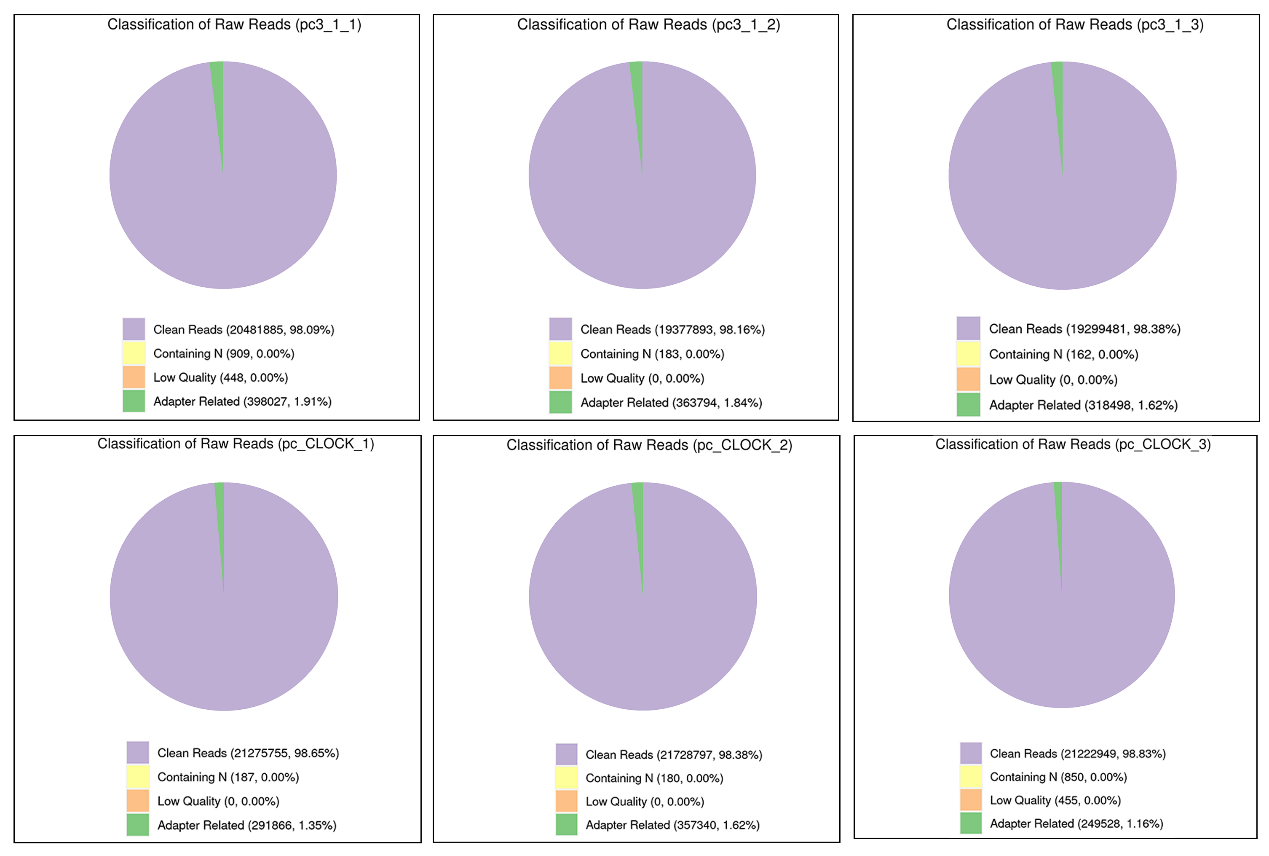


**Fig. S7** The number of reads in transcriptome sequencing


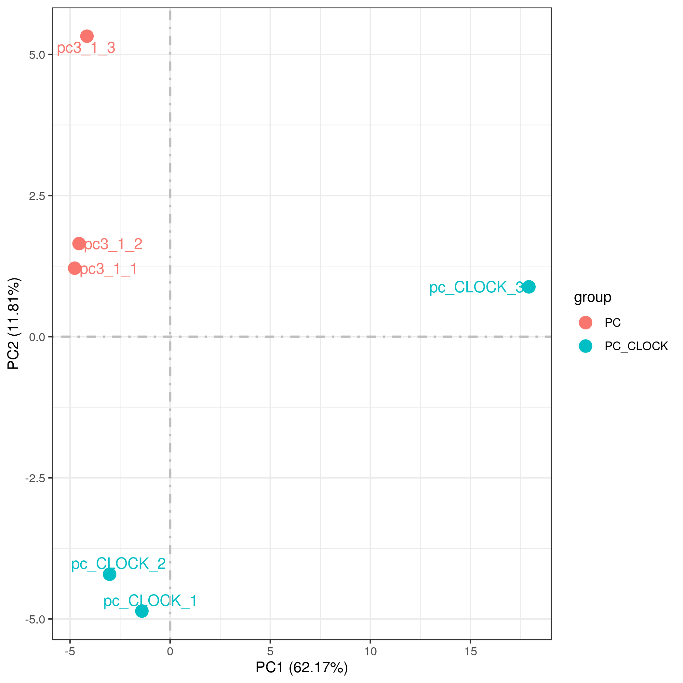


**Fig. S8** Principal components analysis (PCA) in transcriptome sequencing


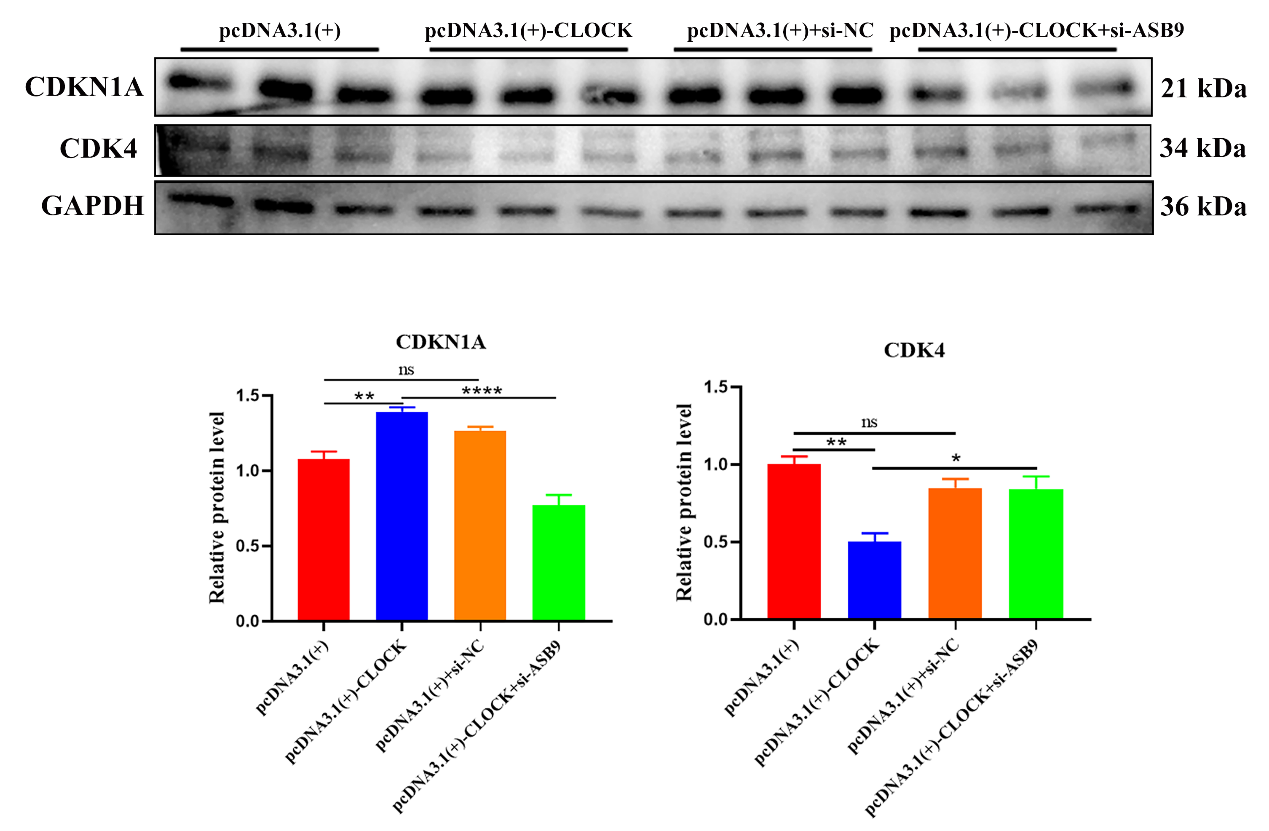


**Fig. S9** A direct association between CLOCK and ASB9 using co-transfection experiments
